# Genetic Variation of the β-tubulin Gene of *Babesia caballi* Strains

**Published:** 2017-09-08

**Authors:** María Guadalupe Montes-Cortés, José Luis Fernández-García, Miguel Ángel Habela Martínez-Estéllez

**Affiliations:** 1 Parasitology and Parasitological Diseases, Veterinary Faculty, Universidad de Extremadura, Cáceres, Spain; 2 Genetics and Animal Breeding, Veterinary Faculty, Universidad de Extremadura, Cáceres, Spain

**Keywords:** *Babesia caballi*, β-tubulin gene, Equine piroplasmosis, PCR-RFLP marker

## Abstract

**Background:**

Equine piroplasmosis is caused by two haemoprotozoan parasites: *Babesia caballi* and *Theileria equi*. Negative economic impact on international trade has been associated to endemic sites. This is the reason why carrier detection requires reliable diagnostic methods. Various diagnostic modalities can be used alone or in combination including PCR. However, genetic variation of commonly used genes is still of debate. The aim of this research was to sequence the β-tubulin gene of a *B. caballi* strain from Spain and to compare it with known β-tubulin sequences.

**Methods:**

DNA was isolated from a cryopreserved strain from Spain and acute and chronic carrier horses. Firstly, degenerated primer pairs were designed based on GenBank sequences of different *Babesia* and *Theileria* species for sequencing. The primers were redesigned to amplify both parasites, simultaneously. Finally, a species-specific primer pair for *B. caballi* was designed and a Restriction Fragment Length Polymorphism-PCR (PCR-RFLP) assay performed to know the difference of known *B. caballi* strains.

**Results:**

We provided new insights of the β-tubulin gene and a good molecular coverage of this gene, contributing with a number of useful primers to amplify *T. equi* and *B. caballi*. Moreover, PCR-RFLP assays based on the exon II of this gene confirmed the causative *B. caballi* strain in Spanish horses.

**Conclusion:**

We reported useful primer pairs for diagnostic and a new sequence of the β-tubulin gene of *B. caballi*, which will facilitate the development of future assays and the detection of infected horses, preventing thus the spread of this disease worldwide.

## Introduction

Equine piroplasmosis is caused by two different haemoprotozoan parasites: *Babesia caballi* (1) and *Theileria equi* (formerly *Babesia equi* Laveran 1901) (2). Both species are transmitted to horses by ticks found in tropical and subtropical regions (3). These parasites cause disease characterized by fever, anaemia, jaundice, weakness, loss of weight, blood urine, oedema, lymphadenopathy and hepatomegaly. Moreover, after infection, horses may remain life-long carriers when they are infected of *T. equi*, whereas horses may remain carriers of *B. caballi* from 1 to 4yr (4). In spite of this difference, both parasitaemia have a serious negative impact on international trade (5), with southern Europe particularly affected as it is an endemic zone of this disease (5–6).

Detection of chronic carriers of Equine piroplasmosis represents a problem for the equine industry, due to the limitations of the available diagnostic methods. Therefore, various diagnostic modalities can be used alone or in combination to diagnose infections (7). These methods are indirect methods such as ELISA and the indirect fluorescent antibody test (IFAT) or direct diagnosis such as microscopic examination of blood smears or PCR (8–7, 9).

Classical single PCR (6), multiplex PCR (10–11), nested PCR (12, 13–14) or real-time PCR (15) are used to diagnose Equine piroplasmosis. Oligonucleotides used in PCR were designed based on genes such us 18S rRNA (6-13-10-15) or equi merozoite antigen gene -*ema*1- (12–16) to amplify *T. equi* genes. On the other hand, to diagnose *B. caballi*, genes such as 18S rRNA (6–10) or the rhoptry-associated protein gene -rap1- (17) are commonly used.

The β-tubulin gene is one of the genes used to diagnose Piroplasmids (18), although its use is not widespread in the detection of *B. caballi* because little is yet known about the genetic variability of this gene in this species, the proof of this is that there is only genetic information from one sequence in data bases (Acc. Nº: AJ289246)

The prevalence of *B. caballi* in horses in our country ranges from 7.9% (García-Bocanegra et al. 2013) to 21.3% (20). Whereby, the detection of chronic carriers is a prime goal to prevent the spread of this disease, which can be achieved by increasing the knowledge about genetic variation of the β-tubulin gene with differential diagnostic purposes.

The aim of this research was to sequence the β-tubulin gene from one isolate of *B. caballi* from Spain by developing primers using β-tubulin genes deposited in Gene Bank from different haemoprotozoan species, to compare it with one known sequence of this gene from *B. caballi* and to provide markers suitable for diagnosis and molecular epidemiology.

## Materials and Methods

### Blood samples

**Fig. 1.**
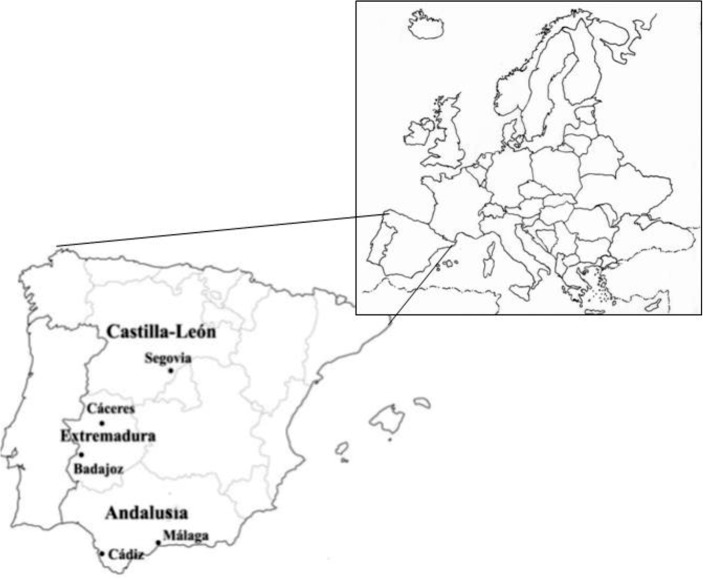
Geographical distribution of collected samples

All the blood samples used in this research were collected from the jugular vein into sterile vacuum tubes (vacutainer®) with and without anticoagulant (EDTA) and routinely thin blood smears were stained and were examined under a microscope at 1500× magnification. *Babesia caballi* was detected in the blood sample from Málaga (Andalusia, Spain) which was sent to our laboratories in Cáceres in 2006. This parasite was cultured in equine erythrocytes (21) and cryopreserved in liquid nitrogen, which still belongs to our sample bank (named GM Malaga strain). This isolated was used to obtain template DNA for sequencing the β-tubulin gene. Moreover, blood samples of acute infected and chronic carrier horses were collected from different regions of centre and southwest Spain in 2011 as follows: five samples from acute infected horses (Cáceres province) and samples from chronic carriers from Segovia (n=5), Cádiz (n=7), Badajoz (n=6) and Cáceres (n=2) (Fig. 1). All the acute cases were positive by microscopic examination but chronic carriers were negative to this procedure. These last blood samples were checked by IFAT (22) and by commercial cELISA test (kits from VMRD Inc. Pullman, WA, USA), being all of them *B. caballi* positive to both techniques (Fig. 1).

### DNA extraction

Whole genomic DNA was obtained from the cryopreserved strain, five samples from acute infected horses and 20 blood samples collected from chronic carriers, tested by cELISA to assess them as carriers of *B. caballi* parasite. Forty μL or 250μL of culture fluid or red cell, respectively, were processed by a proteinase K/salting out procedure (23–24) modified using Zymo-Spin IIC columns (Zymo Research Corporation, Irvine, CA 92614, USA) to purify nucleic acids. Genomic DNA was semi quantitatively analysed using Lambda DNA Marker (λ DNA-HindIII Digest, New England Biolabs®inc.) by visualization in 0.8% agarose gel electrophoresis stained with ethidium bromide following manufacturer recommendations.

### Primer design and PCR amplification

Several available complete or partial sequences of the β-tubulin gene were downloaded from the Gene Bank (Table 1). These sequences, aligned using MEGA4 software (25), aimed at finding a conserved segment where universal PCR primers could be designed to amplify the longest fragment of this gene by conventional PCR.

**Table 1.** Sequences used to design primers

**Accession number**	**Species**	**Gene**	**Chromosome**	**Nucleotide positions or size**	**Author**
**CP001669**	*Babesia equi*	Complete genome	1	845478…846856	Kappmeyer et al. 2012
**AAGK01000002**	*Theileria parva*	Complete genome	2	923513…925358	Gardner et al. 2005.
**AJ289249**	*Babesia equi*	Partial	--	349bp	Cacciò et al. 2000
**XM-001611566**	*Babesia bovis*	Complete cds	--	1343bp	Brayton et al. 2007
**KC465972**	*Babesia odoicoilei*	Partial cds	--	1275bp	Zamoto-Niikura et al. 2014
**AB083377**	*Babesia microti*	Complete cds	--	1651bp	Zamoto et al. 2004
**AB860327**	*Babesia ovata*	Partial cds	--	457bp	Damdinjav et al. unpublished
**AP011947**	*Theileria orientalis*	Complete genome	2	933579…934685	Hayashida et al. 2012
**AJ289246**	*Babesia caballi*	partial	--	460bp	Cacciò et al. 2000

All aligned stretches which expanded from nucleotide 845478 (ie, −2 nucleotide from start codon) to nucleotide 846287 regarding sequence CP001669 were selected for primers design. Because completely conserved segments were not found at both endpoints, useful primers, which included degenerate nucleotides, were obtained from this alignment as follows: B-Tub F and reverse B-Tub R (Table 2).

**Table 2.** Oligonucleotide primers used to amplify and sequence parasite β-tubulin gene.

**Primer Pair**	**Forward Sequence 5′-3′ (F)**	**Reverse Sequence 5′-3′ (R)**	***B. caballi* Amplicon size**	***T. equi* Amplicon size**
**B-Tub**	GAATGAGRGARATCGTWCACA	CARCTTYAGNGTNCKRAAGCARA	826bp	Not done
**B-Tub 2**	GAATGAGGGARATCGTWCACA	CAGCTTTAGRGTTCKGAAGCARAT	826bp	718
**GM B-Tub**	ACCCGGTAAGTCGTTAAACC	AGTTGTCRGGYCTGAAGAGT	345bp	No amplification

The fragment of β-tubulin gene of *B. caballi* was obtained by PCR based on these degenerate primers (which included nucleotides ambiguities, Table 2). In the first step, DNA from *in vitro* cultured *B. caballi* strain was used. Duplicated PCR were carried out in 50μL reaction volumes with 5μL of DNA template and 45μL mixture containing final concentration of 1X PCR buffer, 0.2mM dNTPs, 1pmol each primer (B-Tub R and F or B-Tub R2 and F2 depending on the desired reaction), 1.5MgCl_2_ and 1 unit of Taq DNA polymerase (Ecogen®) on a thermal cycler 2720 (Life Technologies^®^). To maximize amplification success with these degenerate primers a modified-touchdown program was used as follows: (1) each of the first four cycles were run at different annealing temperatures ranging from 55 °C to 51 °C and (2) the remaining 37 cycles at 52 °C. The general amplification conditions were: 96 °C for 5min, then each cycle at 96° for 30s, primer annealing (see (1) above) for 30s and 72 °C for 1min. extension, finally 72 °C for 7min. Five μL of products for both PCR and, later, PCR-RFLP were always run in 1.5% LM agarose gels (Low Melting agarose) buffered on 0.5% TBE with power supply limited to 120v for 35–45min according the expected fragment size and stained with ethidium bromide for checking. PCR products obtained from the Málaga isolated were further used for sequencing.

After sequencing the PCR product and realigning it to those in Table 2, a less degenerated primer pair was designed but in such way that it was able to amplify both the DNA template from *B. caballi* and *T. equi* simultaneously, since both species can be found in infected horses. The new designed primers were as follows: B-Tub F2 and B-Tub R2 (Table 2). The PCR conditions for this assay were: 94 °C for 5min, 94 °C 45s, primer annealing for 52 °C for 1min and 72 °C for 1min extension with 37 cycles, finally extension 72 °C for 7min. Reaction volumes consisted of 1μL of template and 19μL mixture containing final concentration of 1X PCR buffer, 0.2mM dNTPs, 1pmol each primer, 1.5MgCl_2_ and 1 unit of Taq DNA polymerase (Ecogen®) on a thermal cycler 2720 (Life Technologies^®^).

### DNA sequencing and resequencing

PCR product from the cultured isolate from Málaga was purified with ExoSAP-IT (GE Healthcare^®^). Sequencing and resequencing was performed to determine the genomic variations by the BigDye Terminator v.3.1 Cycle Sequencing Kit in both directions using B-Tub F2 and B-Tub R2, respectively (26).

### Species-specific primers for *Babesia caballi*

Three primer pairs were designed to amplify the β-tubulin gene of *B. caballi* from genomic DNA of cryopreserved strain. But the primer pair GM B-Tub F and GM B-Tub R (Table 2), which expanded from the start of the first intron to partially the second exon, was designed based on both sequences (this research and Acc. N. AJ289246) to specifically amplify *B. caballi*. Its specificity was studied using template DNA from *T. equi*, which showed no amplification (data not showed).

### Restriction fragment length polymorphism analysis (PCR-RFLP)

To test the *B. caballi* strain described here a PCR-RFLP analysis was used. The PCRRFLP assays were designed to discriminate both known sequences using software simulation. Using PROPHET 5.0 software (BBN Systems and technologies) a restriction mapping analysis was performed to predict restriction enzymes useful to discriminate between both known strains of *B. caballi* (the Spanish isolated Acc. N. KX358867 and the French one with Acc. N. AJ289246). Based on simulation of cleavage sites, two restriction enzymes, HaeIII and HinfI, were selected. The cutting prediction for each strain can be seen in Table 3.

**Table 3.** Restriction mapping analysis for predicting restriction enzymes. (*excluding cuts within primers)

***B. caballi* strain**	**Size of PCR product**	**Predicted fragment sizes**	
		**H*ae*III (GG↓CC)**	**H*inf*I (G↓ANTC)**
**Cacciò et al. (2000)**	347	249,88 *	299,48
**This research**	345	Uncut *	297,48

## Results

**Fig. 2.**
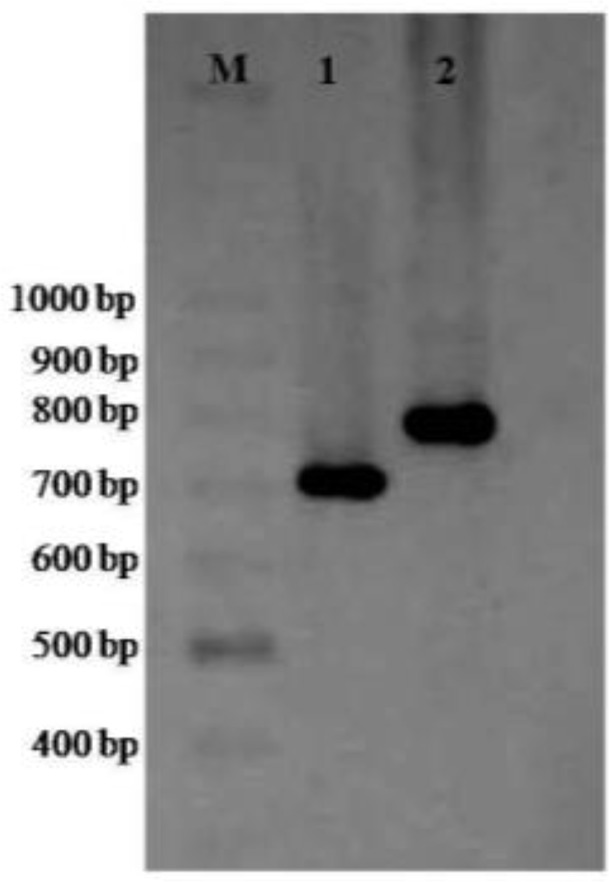
Amplicons of *Theileria equi* and *Babesia caballi* using primers pairs B-Tub 2. Lane M, 100bp size marker. Lane 1: Amplicon of *T. equi* (718bp) Lane 2: Amplicon of *B. caballi* (826bp)

This research provided a new sequence of the β-tubulin gene of *B. caballi* (GM Málaga strain). This DNA stretch was sufficiently long for a successful primer pairs design. As a result of comparing the β-tubulin gene of the two most important piroplasms species that impair horses, *T. equi* and *B. caballi*, a first primer pair (B-TubF and B-TubR, Table 2) was designed in order to amplify that gene from a DNA template of both parasites. Moreover, new and less degenerated primers pair could be designed (B-TubF2 and B-TubR2, Table 2) used for conventional PCR applications. The resulted amplicons had a size of 718bp and 826bp for *T. equi* and *B. caballi*, respectively (Fig. 2).

According to (27), the β-tubulin gene sequences from each of the two species differed in length (100bp approx.). The sequence of the β-tubulin gene of *B. caballi* obtained with these primers provided more molecular information about the genetic variations of this conserved gene.

**Fig. 3.**
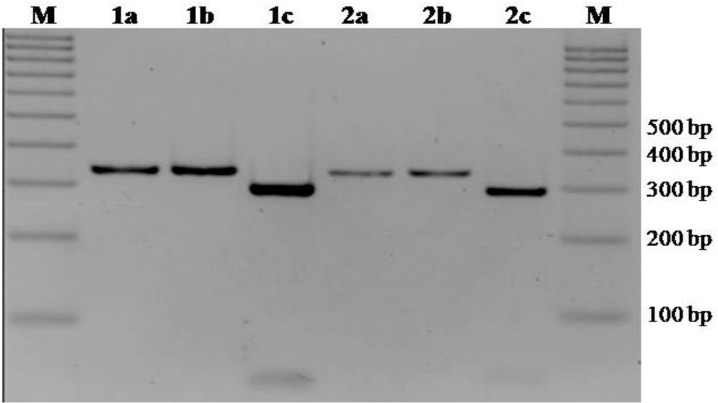
Restriction enzymes assays. Lane M, 100bp size marker. Lane 1a: PCR product of DNA from cultivated isolate amplified with primers pairs GM BTub. Lane 1b: PCR product digested with HaeIII (uncut). Lane 1c: PCR product digested with HinfI. Lane 2a: PCR product of DNA from an acute infected horse with primers pairs GM B-Tub. Lane 2b: PCR product digested with HaeIII (uncut). Lane 2c: PCR product digested with HinfI

Both sequences (AJ289246 and KX358867) were truncated to accommodate to the shortest molecule, i.e., 460bp. Thus, this fragment of the β-tubulin gene of *B. caballi* partly covered exon I (nucleotide 1 to 63), but completely covered intron I (nucleotide 64 to 233) and exon II (nucleotide 234 to 460). Sixty-eight polymorphic sites were found between both sequences. The comparison of both sequences using BLASTN 2.2.20 (28) yielded 81% nucleotides identities and 3% open gaps. Focusing on exon II, they shared 92% identities with 19 (19 of 76 triples) silent mutations (synonymous substitutions), as estimated using the DnaSP software version 4.0. This analysis reported an unexpected number of polymorphisms between both strains at the coding region but without changing the primary structure of the protein, as should be in the case of strongly conserved genes such as the β-tubulin gene (27). Moreover, the first intron showed a minor size variation due to a 2bp deletion in the Spanish sequence. All these facts suggest the sequence of β-tubulin gene could be variable among and within species of *Babesia* parasites. Twenty DNA samples from chronic carriers and 5 samples from acute infected horses were amplified using our species-specific primers for *B. caballi*. All samples were positive. These PCR were digested with both restriction enzymes (HaeIII and Hinf I) to determine the strain causing the infection. HinfI cut the PCR amplicons but HaeIII does not cut them as was predicted. As a result, this assay was able to confirm that the infecting strain of these horses would be the Spanish strain (Fig. 3).

## Discussion

This research has provided new insights into the β-tubulin gene of *B. caballi*, the key factor being that there was a unique and partial sequence available in the Gene Bank for this piroplasm. This sequence belonged to a different *B. caballi* strain after comparison with the sequence from (27). Moreover, it can be suggested that genetic variation should be expected among *B. caballi* strains even for conserved genes. The occurrence of genetic variations in certain conserved genes like the β-tubulin gene (described in this research), could indicate not only the existence of different strains of *B. caballi*, but also suggests the study of possible higher taxonomic levels because in our study the shared identity was within the 92% for coding segments. Similar results were recently reported for the β-tubulin gene sequence of *B. ovata.* This *Babesia* shared 94.8–100% identity within species but the shared sequence identity of *B. ovata* with other species was 87.0–90.6%, 83.7–86.3%, and 82.7–85.4% to *B. bigemina*, *B. odocoilei* and *B. divergens*, respectively (29).

Based on genetic knowledge of 18S rRNA gene both for *B. caballi* and *T. equi* (30–31) there were different genotypes in Spain for both species. On the one hand, (32) conducted phylogenetic analysis based on the same gene to report that there were two *B. caballi* clades. However, (33) established four *B. caballi* clades using the BC48 gene. On the other hand, (32) found three *T. equi* clades. However, (34) announced that there were not three, but four *T. equi* clades. Phylogenetic analysis by (33) using the ema-1 gene divided the sequences into four *T. equi* clades. (35) using a phylogenetic study based on both the 18S rRNA gene and the β-tubulin gene have shown *T. annae* and its synonyms are not *Theileria* parasites instead they are *Babesia* species, being the β-tubulin decisive to determine that reclassification.

This information suggests that taxonomic classification of equine piroplasms is not yet defined completely as reported (3). According to (36) and (37), a new diagnostic test on additional or new antigens is needed because nowadays there is not a gold standard test for detecting Equine piroplasmosis. Specifically, (37) showed there was lack of concordance between the *B. caballi* cELISA, IFAT, and nPCR. Hence, the importance of this study that contributes to the enrichment of current genetic information of an unknown β-tubulin gene of *B. caballi*.

Based on the PCR-RFLP analysis, it was possible to discriminate different piroplasms species as shown by (27) where cattle piroplasms such as *Theileria sergenti*, *T. annulata*, *B. bigemina*, *B. bovis* and *B.a major* were efficiently discriminated using R*sa*I restriction enzyme. Another study also valued PCRRFLP as a specific analysis to confirm species diagnosis as has been shown for *B. bigemina* with V*sp*I digestion of the small subunit rRNA. Using B*sl*I and H*inf*I with PCR amplicons from the 18S ribosomal RNA gene, discriminated subspecies of canine piroplasms from other species such as *T. equi*, *T. annae* and *B. conradae*.

PCR-RFLP assays may not be necessary to discriminate between *B. caballi* and *T. equi* species due to the different sizes of their intron I (27 and this study). However, this difference in size was as short as 2bp between both *B. caballi* strains, which cannot be seen with agarose gels. This is the reason why PCR-RFLP assays were selected to discriminate the GM Málaga strain from the strain described by (27), so this study shows for the first time that PCR-RFLP assays were used in order to discriminate among different strains of *B. caballi* species assuming predictions based on the sequence deposited in the Gene Bank. Moreover, although PCR-RFLP compares a limited number of nucleotides, restriction enzyme digestion was in full accordance with the predicted target based on the sequence of the GM Málaga strain even when using DNA from naturally infected horses.

Finally, this study has provided several new and useful primer pairs although they should be more deeply assessed for diagnostic purposes (acute infected horses and chronic *B. caballi* carriers) in future assays.

## Conclusion

We reported here that the β-tubulin gene of *B. caballi* shows high genetic variation, which will be useful to facilitate the development of more precise molecular assays for the detection of infected horses. This is equally relevant at local and international levels, helping to prevent the spread of this disease worldwide in the future.
